# 2-({[(2*S*)-1-Hy­droxy-1,1,3-tri­phenyl­propan-2-yl]imino}­meth­yl)-4,6-bis­(4-methyl­phen­yl)phenol

**DOI:** 10.1107/S241431462001576X

**Published:** 2020-12-04

**Authors:** Veronica L. Show, Emily Y. Fok, Adam R. Johnson

**Affiliations:** a Department of Chemistry, Harvey Mudd College, 301 Platt Blvd., Claremont, CA 91711, USA; University of Otago, New Zealand

**Keywords:** crystal structure, Schiff base, tridentate ligand, chiral

## Abstract

The title compound, C_42_H_37_NO_2_, crystallizes in the ortho­rhom­bic space group *P*2_1_2_1_2_1_ with one mol­ecule in the asymmetric unit. An intra­molecular hydrogen bond orients the phenol hydroxyl group toward the imine nitro­gen. The aliphatic alcohol is engaged in a weak intra­molecular hydrogen bond with the imine nitro­gen.

## Abstract 2

The title compound was prepared and fully characterized. The salicyl­aldehyde alcohol is engaged in an intra­molecular O—H⋯N hydrogen bond with the imine nitro­gen.

## Graphical abstract

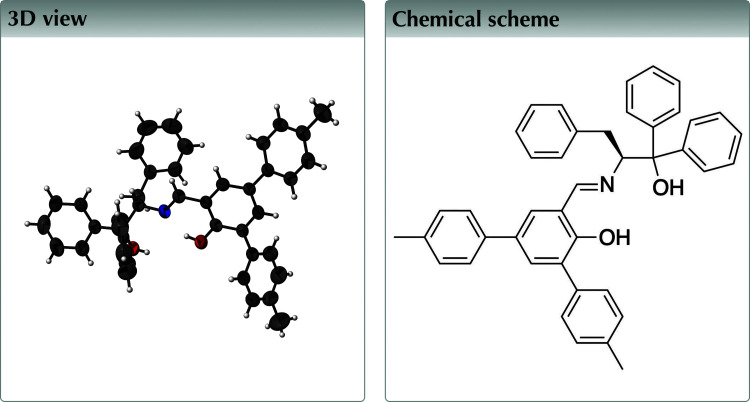

## Structure description

We have synthesized a number of chiral imine diols by Schiff-base condensation of the corresponding salicyl­aldehydes with (*S*)-2-amino-1,1,3-tri­phenyl­propanol (Kang *et al.*, 2004 ▸; Liu *et al.*, 2004 ▸). These compounds serve as ligands for titanium for the asymmetric intra­molecular hydro­amination of amino­allenes (Sha *et al.*, 2019 ▸; Fok *et al.*, 2020 ▸). The absolute structure parameter of 0.1 (7) has a large uncertainty but the absolute configuration was verified by synthesis and polarimetry.

**Figure 1:**
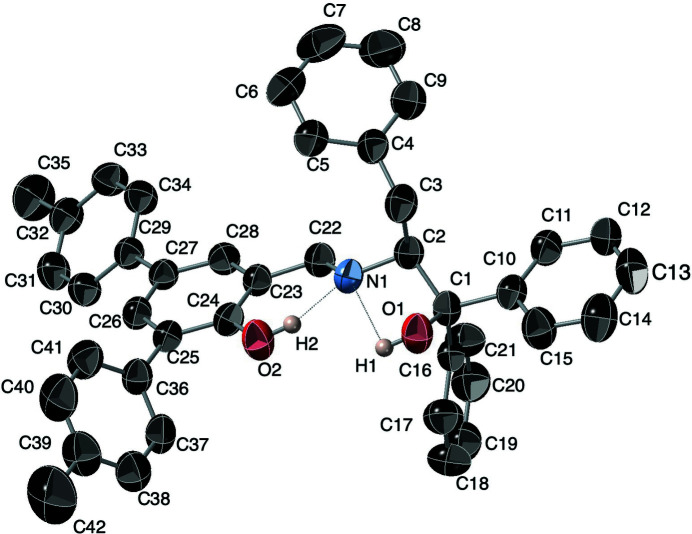
The asymmetric unit of the title compound, showing the atom-numbering scheme. The displacement ellipsoids are shown at the 50% probability level. Hydrogen atoms apart from H1 and H2 have been omitted for clarity and intramolecular hydrogen bonds are shown as dashed lines. Figure generated using *CrystalMaker* (Palmer, 2020 ▸).

The compound reported here has the expected imine–phenol structure (Fig. 1 ▸) as opposed to the iminium–phenoxide tautomer seen in derivatives with less steric bulk. There is an intra­molecular O2—H2⋯N1 hydrogen bond, Table 1 ▸. The phenol aromatic ring (C23–C28) is essentially co-planar with O2, C22, and N1; O2 is 0.046 (3) Å above the plane, C22 is 0.083 (4) Å above the plane, and N1 is 0.180 (3) Å above the plane. The C22—N1—C2—C1 torsion angle is 136.5 (3)°, and the N1—C2—C1—O1 torsion angle is 60.2 (3)°, and these result in the positioning of C1 and H1 being pointed towards the open space near N1, and a intra­molecular O1—H1⋯N1 hydrogen bond. O1 becomes almost coplanar with the phenol ring, only 0.198 (3) Å below the plane.

Unlike the significant bond alternation seen in related structures (Sha *et al.*, 2019 ▸), the bonds within the phenol ring (C23–C28) are all between 1.391 (4) and 1.406 (4) Å. The aromatic rings on the benzyl and phenyl substituents have typical aromatic bond distances ranging from 1.34–1.41 Å. The aromatic C24—O2 bond at 1.354 (4) Å is substanti­ally shorter than the aliphatic C1—O1 bond of 1.435 (4) Å, as seen in related structures.

## Synthesis and crystallization

**Figure 2:**
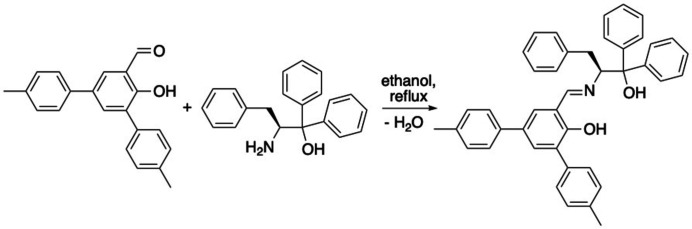
Synthesis of the title compound.

Details of the preparation of the title compound are shown in Fig. 2 ▸. 2-Hy­droxy-3,5-di-4-(meth­yl)phenyl­benzaldehyde (0.8654 g, 2.86 mmol) and (*S*)-2-amino-1,1,3-tri­phenyl­propanol (0.8651 g, 2.86 mmol, 1 equiv.) were dissolved in ethanol (50 ml) and heated overnight at reflux. The solvent was removed *in vacuo*. The crude product was purified by flash column chromatography to yield an orange solid (1.348 g, 1.52 mmol, 80.1%). X-ray quality crystals were obtained by slow evaporation of a toluene solution. M.p. 107.7–108.4°C. [α]_D_: −165° (*c =* 0.006 g ml^−1^, EtOAc). Analysis calculated for C_42_H_37_NO_2_: C, 85.83; H, 6.34; N, 2.38. Found: C, 85.47; H, 6.43; N, 2.35. ^1^H NMR (400 MHz, CDCl_3_): 13.22 (*s*, 1H, ArO*H*), 7.72–6.99 (*m*, 26H, Ar*H*, *H*C=N), 4.41 (*dd*, 1H, *J* = 10.0, 1.6 Hz, C*H*CH_a_H_b_Ph), 3.02 (apparent *d*, 1H, *J* = 12.5 Hz, CHC*H_a_* H_b_Ph), 2.96 (*s*, 1H, O*H*), 2.89 (*dd*, 1H, *J* = 13.8, 10.2 Hz, CHCH_a_ *H_b_* Ph), 2.41 (*s*, 3H, CH_3_), 2.38 (*s*, 3H, CH_3_). ^13^C NMR (100 MHz, CDCl_3_): 167.11 (H*C=*N), 157.34 (4°), 145.66 (4°), 144.20 (4°), 139.06 (4°), 137.42 (4°), 137.23 (4°), 136.76 (4°), 134.67 (4°), 132.07 (4°), 131.95 (CH), 130.03 (4°), 129.88 (CH), 129.64 (CH), 129.34 (CH), 129.18 (CH), 129.10 (CH), 128.64 (CH), 128.56 (CH), 128.51 (CH), 128.37 (CH), 127.18 (CH), 127.06 (CH), 126.53 (CH), 126.20 (CH), 125.97 (CH), 118.74 (4°), 79.84 (4°), 78.97 (CH, chiral center), 37.54 (CH_2_), 21.37 (CH_3_), 21.18 (CH_3_). MS (APCI): *m*/*z* 589 [*M*+H]^+^. IR (ATR, diamond): (C=N) = 1628 cm^−1^.

## Refinement

Crystal data, data collection and structure refinement details are summarized in Table 2 ▸.

## Supplementary Material

Crystal structure: contains datablock(s) I. DOI: 10.1107/S241431462001576X/sj4216sup1.cif

Structure factors: contains datablock(s) I. DOI: 10.1107/S241431462001576X/sj4216Isup2.hkl

Supporting information file. DOI: 10.1107/S241431462001576X/sj4216Isup3.cml

CCDC reference: 2042781

Additional supporting information:  crystallographic information; 3D view; checkCIF report

## Figures and Tables

**Table 1:** Hydrogen-bond geometry (Å, °)

*D*—H⋯*A*	*D*—H	H⋯*A*	*D*⋯*A*	*D*—H⋯*A*
O2—H2⋯N1	0.82	1.80	2.539 (4)	148
O1—H1⋯N1	0.82	2.46	2.763 (3)	103

**Table 2:** Experimental details

Crystal data	
Chemical formula	C_42_H_37_NO_2_
*M*_r_	587.72
Crystal system, space group	Orthorhombic, *P*2_1_2_1_2_1_
Temperature (K)	293
*a*, *b*, *c* (Å)	9.2554 (3), 11.5721 (4), 31.9214 (10)
*V* (Å^3^)	3418.92 (19)
*Z*	4
Radiation type	Mo *K*α
μ (mm^−1^)	0.07
Crystal size (mm)	0.31 × 0.16 × 0.11

Data collection	
Diffractometer	XtaLAB Mini II
Absorption correction	Analytical [*CrysAlis PRO* (Rigaku OD, 2019 ▸) and ABSPACK (Rigaku OD, 2017 ▸)]
*T*_min_, *T*_max_	0.982, 0.994
No. of measured, independent and observed [*I* > 2σ(*I*)] reflections	88879, 6086, 3835
*R*_int_	0.074
(sin θ/λ)_max_ (Å^−1^)	0.597

Refinement	
*R*[*F* ^2^ > 2σ(*F* ^2^)], *wR*(*F* ^2^), *S*	0.052, 0.112, 1.01
No. of reflections	6086
No. of parameters	410
H-atom treatment	H-atom parameters constrained
Δρ_max_, Δρ_min_ (e Å^−3^)	0.12, −0.11
Absolute structure	Flack *x* determined using 1229 quotients [(*I* ^+^)-(*I* ^-^)]/[(*I* ^+^)+(*I* ^-^)] (Parsons, *et al*, 2013 ▸)
Absolute structure parameter	0.1 (7)

Computer programs: *CrysAlis PRO* (Rigaku OD, 2019 ▸), *SHELXT* (Sheldrick, 2015*a* ▸), *SHELXL* (Sheldrick, 2015*b* ▸), *OLEX2* (Dolomanov *et al.*, 2009 ▸) and *CrystalMaker* (Palmer, 2020 ▸).

## full crystallographic data

### Crystal data

**Table:**

C_42_H_37_NO_2_	*D*_x_ = 1.142 Mg m^−^^3^
*M**_r_* = 587.72	Mo *K*α radiation, λ = 0.71073 Å
Orthorhombic, *P*2_1_2_1_2_1_	Cell parameters from 14764 reflections
*a* = 9.2554 (3) Å	θ = 1.9–20.8°
*b* = 11.5721 (4) Å	µ = 0.07 mm^−^^1^
*c* = 31.9214 (10) Å	*T* = 293 K
*V* = 3418.92 (19) Å^3^	Block, clear light yellow
*Z* = 4	0.31 × 0.16 × 0.11 mm
*F*(000) = 1248	

### Data collection

**Table:**

XtaLAB Mini II diffractometer	6086 independent reflections
Radiation source: fine-focus sealed X-ray tube, Rigaku (Mo) X-ray Source	3835 reflections with *I* > 2σ(*I*)
Graphite monochromator	*R*_int_ = 0.074
Detector resolution: 10.0000 pixels mm^-1^	θ_max_ = 25.1°, θ_min_ = 2.2°
ω scans	*h* = −11→11
Absorption correction: analytical [CrysAlisPro (Rigaku OD, 2019) and ABSPACK (Rigaku OD, 2017)]	*k* = −13→13
*T*_min_ = 0.982, *T*_max_ = 0.994	*l* = −38→38
88879 measured reflections	

### Refinement

**Table:**

Refinement on *F*^2^	Hydrogen site location: inferred from neighbouring sites
Least-squares matrix: full	H-atom parameters constrained
*R*[*F*^2^ > 2σ(*F*^2^)] = 0.052	*w* = 1/[σ^2^(*F*_o_^2^) + (0.0556*P*)^2^ + 0.0355*P*] where *P* = (*F*_o_^2^ + 2*F*_c_^2^)/3
*wR*(*F*^2^) = 0.112	(Δ/σ)_max_ = 0.001
*S* = 1.01	Δρ_max_ = 0.12 e Å^−^^3^
6086 reflections	Δρ_min_ = −0.11 e Å^−^^3^
410 parameters	Absolute structure: Flack *x* determined using 1229 quotients [(*I*^+^)-(*I*^-^)]/[(*I*^+^)+(*I*^-^)] (Parsons, *et al*, 2013)
0 restraints	Absolute structure parameter: 0.1 (7)
Primary atom site location: dual	

### Special details

**Table:**

Geometry. All esds (except the esd in the dihedral angle between two l.s. planes) are estimated using the full covariance matrix. The cell esds are taken into account individually in the estimation of esds in distances, angles and torsion angles; correlations between esds in cell parameters are only used when they are defined by crystal symmetry. An approximate (isotropic) treatment of cell esds is used for estimating esds involving l.s. planes.

### Fractional atomic coordinates and isotropic or equivalent isotropic displacement parameters (Å^2^)

**Table:**

	*x*	*y*	*z*	*U*_iso_*/*U*_eq_	
O1	0.6294 (3)	0.4955 (2)	0.24453 (7)	0.0653 (7)	
H1	0.689147	0.504113	0.263236	0.098*	
O2	0.7917 (3)	0.4887 (2)	0.35057 (7)	0.0692 (7)	
H2	0.736655	0.464313	0.332477	0.104*	
N1	0.6319 (3)	0.3457 (2)	0.31194 (8)	0.0532 (7)	
C1	0.6062 (4)	0.3744 (3)	0.23755 (10)	0.0517 (9)	
C2	0.5320 (4)	0.3249 (3)	0.27742 (9)	0.0504 (9)	
H2A	0.516244	0.241633	0.274077	0.060*	
C3	0.3884 (4)	0.3847 (3)	0.28819 (10)	0.0639 (10)	
H3A	0.407654	0.463637	0.296750	0.077*	
H3B	0.328159	0.387332	0.263330	0.077*	
C4	0.3080 (4)	0.3237 (3)	0.32255 (11)	0.0556 (9)	
C5	0.3318 (5)	0.3464 (4)	0.36421 (12)	0.0798 (13)	
H5	0.396579	0.404315	0.371609	0.096*	
C6	0.2621 (6)	0.2855 (5)	0.39534 (14)	0.0977 (16)	
H6	0.280074	0.303258	0.423272	0.117*	
C7	0.1695 (6)	0.2017 (4)	0.38588 (17)	0.0970 (16)	
H7	0.124816	0.159862	0.407115	0.116*	
C8	0.1405 (6)	0.1773 (4)	0.34539 (19)	0.1091 (17)	
H8	0.074177	0.119804	0.338752	0.131*	
C9	0.2096 (5)	0.2380 (4)	0.31360 (14)	0.0921 (14)	
H9	0.188961	0.220341	0.285810	0.111*	
C10	0.5138 (4)	0.3654 (3)	0.19787 (10)	0.0543 (9)	
C11	0.4240 (4)	0.2731 (3)	0.18940 (11)	0.0677 (11)	
H11	0.414767	0.214442	0.209129	0.081*	
C12	0.3474 (4)	0.2657 (4)	0.15224 (13)	0.0770 (12)	
H12	0.285473	0.203753	0.147597	0.092*	
C13	0.3629 (5)	0.3501 (4)	0.12213 (14)	0.0828 (13)	
H13	0.309935	0.346367	0.097415	0.099*	
C14	0.4574 (5)	0.4401 (4)	0.12889 (13)	0.0870 (14)	
H14	0.471721	0.495095	0.108055	0.104*	
C15	0.5317 (5)	0.4495 (4)	0.16667 (12)	0.0744 (12)	
H15	0.593370	0.511637	0.171223	0.089*	
C16	0.7499 (4)	0.3123 (3)	0.22963 (9)	0.0522 (9)	
C17	0.8731 (5)	0.3723 (4)	0.21882 (12)	0.0772 (12)	
H17	0.870032	0.452549	0.217305	0.093*	
C18	1.0013 (5)	0.3154 (5)	0.21019 (14)	0.0910 (14)	
H18	1.082786	0.357674	0.202763	0.109*	
C19	1.0091 (5)	0.1980 (5)	0.21249 (13)	0.0831 (13)	
H19	1.095450	0.159806	0.206974	0.100*	
C20	0.8883 (5)	0.1375 (4)	0.22298 (12)	0.0778 (12)	
H20	0.891793	0.057265	0.224532	0.093*	
C21	0.7618 (4)	0.1940 (4)	0.23126 (11)	0.0703 (11)	
H21	0.680645	0.150724	0.238279	0.084*	
C22	0.6586 (4)	0.2695 (3)	0.33965 (10)	0.0550 (9)	
H22	0.620090	0.195694	0.336849	0.066*	
C23	0.7494 (4)	0.2969 (3)	0.37590 (10)	0.0496 (9)	
C24	0.8122 (4)	0.4061 (3)	0.38002 (10)	0.0526 (9)	
C25	0.8931 (4)	0.4348 (3)	0.41580 (10)	0.0500 (9)	
C26	0.9079 (4)	0.3519 (3)	0.44648 (10)	0.0529 (9)	
H26	0.959861	0.370856	0.470443	0.064*	
C27	0.8493 (4)	0.2410 (3)	0.44368 (10)	0.0478 (8)	
C28	0.7703 (4)	0.2159 (3)	0.40773 (10)	0.0541 (9)	
H28	0.730155	0.142665	0.404804	0.065*	
C29	0.8755 (4)	0.1531 (3)	0.47686 (10)	0.0504 (9)	
C30	1.0031 (4)	0.1530 (3)	0.49976 (11)	0.0604 (10)	
H30	1.071466	0.210327	0.494851	0.072*	
C31	1.0305 (4)	0.0696 (4)	0.52972 (11)	0.0683 (11)	
H31	1.116596	0.072379	0.544695	0.082*	
C32	0.9326 (4)	−0.0180 (3)	0.53792 (11)	0.0622 (10)	
C33	0.8045 (4)	−0.0174 (3)	0.51594 (11)	0.0621 (10)	
H33	0.735757	−0.074099	0.521325	0.075*	
C34	0.7762 (4)	0.0665 (3)	0.48582 (10)	0.0591 (10)	
H34	0.688924	0.064645	0.471381	0.071*	
C35	0.9668 (5)	−0.1114 (4)	0.56979 (13)	0.0922 (15)	
H35A	0.905661	−0.102056	0.593855	0.138*	
H35B	1.066098	−0.105268	0.578152	0.138*	
H35C	0.950142	−0.185964	0.557520	0.138*	
C36	0.9554 (4)	0.5526 (3)	0.42036 (11)	0.0561 (10)	
C37	1.0498 (4)	0.5958 (3)	0.39045 (11)	0.0650 (10)	
H37	1.074983	0.550160	0.367610	0.078*	
C38	1.1070 (5)	0.7055 (4)	0.39409 (13)	0.0770 (12)	
H38	1.169606	0.732566	0.373533	0.092*	
C39	1.0733 (6)	0.7756 (4)	0.42744 (16)	0.0842 (14)	
C40	0.9821 (6)	0.7310 (4)	0.45696 (15)	0.0924 (14)	
H40	0.958615	0.776139	0.480080	0.111*	
C41	0.9239 (4)	0.6224 (4)	0.45388 (12)	0.0730 (12)	
H41	0.862259	0.595760	0.474761	0.088*	
C42	1.1356 (8)	0.8968 (4)	0.43117 (18)	0.143 (2)	
H42A	1.082749	0.948409	0.413288	0.214*	
H42B	1.235355	0.895979	0.422919	0.214*	
H42C	1.128080	0.922631	0.459675	0.214*	

### Atomic displacement parameters (Å^2^)

**Table:**

	*U*^11^	*U*^22^	*U*^33^	*U*^12^	*U*^13^	*U*^23^
O1	0.090 (2)	0.0512 (15)	0.0548 (15)	−0.0053 (15)	−0.0084 (14)	−0.0028 (12)
O2	0.092 (2)	0.0583 (15)	0.0569 (15)	−0.0119 (14)	−0.0203 (14)	0.0123 (13)
N1	0.0618 (18)	0.0565 (18)	0.0413 (16)	0.0019 (16)	−0.0026 (15)	0.0023 (15)
C1	0.066 (2)	0.045 (2)	0.044 (2)	−0.001 (2)	−0.0056 (19)	−0.0016 (16)
C2	0.056 (2)	0.048 (2)	0.046 (2)	0.0028 (19)	−0.0033 (18)	0.0010 (16)
C3	0.071 (3)	0.068 (2)	0.053 (2)	0.012 (2)	−0.007 (2)	0.0017 (19)
C4	0.055 (2)	0.059 (2)	0.053 (2)	0.010 (2)	−0.0002 (19)	−0.0080 (19)
C5	0.082 (3)	0.093 (3)	0.064 (3)	−0.016 (3)	0.008 (2)	−0.016 (2)
C6	0.104 (4)	0.123 (4)	0.066 (3)	−0.014 (4)	0.024 (3)	−0.006 (3)
C7	0.104 (4)	0.081 (3)	0.106 (4)	0.005 (3)	0.049 (3)	−0.008 (3)
C8	0.109 (4)	0.098 (4)	0.121 (4)	−0.033 (3)	0.041 (4)	−0.039 (4)
C9	0.088 (3)	0.111 (4)	0.077 (3)	−0.018 (3)	0.012 (3)	−0.034 (3)
C10	0.062 (2)	0.053 (2)	0.048 (2)	0.0072 (19)	−0.0011 (18)	−0.0027 (18)
C11	0.078 (3)	0.075 (3)	0.050 (2)	−0.013 (2)	0.000 (2)	0.001 (2)
C12	0.077 (3)	0.097 (3)	0.057 (2)	−0.011 (3)	0.001 (2)	−0.020 (3)
C13	0.086 (3)	0.094 (3)	0.068 (3)	0.017 (3)	−0.022 (3)	−0.018 (3)
C14	0.112 (4)	0.085 (3)	0.064 (3)	0.019 (3)	−0.020 (3)	0.013 (2)
C15	0.090 (3)	0.068 (3)	0.065 (2)	0.000 (2)	−0.017 (2)	0.008 (2)
C16	0.055 (2)	0.061 (2)	0.0408 (19)	−0.007 (2)	−0.0051 (18)	−0.0024 (17)
C17	0.070 (3)	0.083 (3)	0.079 (3)	−0.007 (3)	0.003 (2)	0.008 (2)
C18	0.059 (3)	0.115 (5)	0.099 (4)	−0.016 (3)	0.008 (3)	0.001 (3)
C19	0.069 (3)	0.109 (4)	0.071 (3)	0.018 (3)	−0.001 (2)	−0.009 (3)
C20	0.079 (3)	0.075 (3)	0.080 (3)	0.010 (3)	0.003 (3)	−0.007 (2)
C21	0.062 (3)	0.066 (3)	0.083 (3)	0.000 (2)	0.009 (2)	−0.001 (2)
C22	0.062 (2)	0.054 (2)	0.050 (2)	0.0008 (19)	−0.0030 (19)	0.0009 (18)
C23	0.056 (2)	0.050 (2)	0.0423 (19)	−0.0015 (18)	−0.0014 (17)	0.0007 (17)
C24	0.061 (2)	0.053 (2)	0.044 (2)	0.0029 (19)	0.0006 (18)	0.0043 (17)
C25	0.055 (2)	0.053 (2)	0.0423 (19)	0.0028 (19)	0.0005 (18)	−0.0002 (18)
C26	0.055 (2)	0.061 (2)	0.0427 (19)	0.003 (2)	−0.0013 (17)	−0.0024 (18)
C27	0.050 (2)	0.051 (2)	0.0432 (19)	0.0011 (18)	−0.0006 (17)	0.0037 (16)
C28	0.058 (2)	0.053 (2)	0.052 (2)	−0.0032 (19)	−0.0009 (19)	0.0029 (18)
C29	0.053 (2)	0.054 (2)	0.0452 (18)	0.002 (2)	−0.0029 (19)	0.0027 (17)
C30	0.058 (2)	0.068 (3)	0.055 (2)	−0.005 (2)	−0.0074 (19)	0.009 (2)
C31	0.067 (3)	0.080 (3)	0.058 (2)	0.002 (2)	−0.016 (2)	0.008 (2)
C32	0.071 (3)	0.062 (2)	0.054 (2)	0.008 (2)	−0.003 (2)	0.0097 (19)
C33	0.063 (2)	0.062 (3)	0.062 (2)	−0.003 (2)	0.000 (2)	0.014 (2)
C34	0.058 (2)	0.062 (2)	0.058 (2)	−0.001 (2)	−0.0077 (19)	0.007 (2)
C35	0.108 (4)	0.088 (3)	0.080 (3)	0.011 (3)	−0.016 (3)	0.032 (2)
C36	0.069 (3)	0.051 (2)	0.048 (2)	0.001 (2)	−0.005 (2)	0.0026 (18)
C37	0.090 (3)	0.057 (2)	0.049 (2)	−0.009 (2)	−0.005 (2)	−0.0032 (19)
C38	0.099 (3)	0.068 (3)	0.064 (3)	−0.015 (3)	−0.009 (2)	0.010 (2)
C39	0.112 (4)	0.052 (3)	0.089 (3)	−0.014 (3)	−0.015 (3)	0.002 (3)
C40	0.126 (4)	0.062 (3)	0.089 (3)	−0.001 (3)	0.002 (3)	−0.026 (3)
C41	0.088 (3)	0.063 (3)	0.068 (3)	0.000 (2)	0.009 (2)	−0.013 (2)
C42	0.214 (7)	0.064 (3)	0.149 (5)	−0.046 (4)	−0.014 (5)	−0.014 (3)

### Geometric parameters (Å, º)

**Table:**

O1—H1	0.8200	C20—H20	0.9300
O1—C1	1.435 (4)	C20—C21	1.367 (5)
O2—H2	0.8200	C21—H21	0.9300
O2—C24	1.354 (4)	C22—H22	0.9300
N1—C2	1.459 (4)	C22—C23	1.465 (4)
N1—C22	1.273 (4)	C23—C24	1.397 (4)
C1—C2	1.556 (4)	C23—C28	1.396 (4)
C1—C10	1.532 (5)	C24—C25	1.406 (4)
C1—C16	1.533 (5)	C25—C26	1.378 (4)
C2—H2A	0.9800	C25—C36	1.487 (5)
C2—C3	1.538 (5)	C26—H26	0.9300
C3—H3A	0.9700	C26—C27	1.396 (4)
C3—H3B	0.9700	C27—C28	1.391 (4)
C3—C4	1.501 (5)	C27—C29	1.488 (4)
C4—C5	1.373 (5)	C28—H28	0.9300
C4—C9	1.377 (5)	C29—C30	1.388 (4)
C5—H5	0.9300	C29—C34	1.390 (4)
C5—C6	1.378 (5)	C30—H30	0.9300
C6—H6	0.9300	C30—C31	1.382 (5)
C6—C7	1.329 (6)	C31—H31	0.9300
C7—H7	0.9300	C31—C32	1.385 (5)
C7—C8	1.350 (6)	C32—C33	1.378 (5)
C8—H8	0.9300	C32—C35	1.518 (5)
C8—C9	1.389 (6)	C33—H33	0.9300
C9—H9	0.9300	C33—C34	1.392 (5)
C10—C11	1.379 (5)	C34—H34	0.9300
C10—C15	1.402 (5)	C35—H35A	0.9600
C11—H11	0.9300	C35—H35B	0.9600
C11—C12	1.385 (5)	C35—H35C	0.9600
C12—H12	0.9300	C36—C37	1.388 (5)
C12—C13	1.378 (5)	C36—C41	1.372 (5)
C13—H13	0.9300	C37—H37	0.9300
C13—C14	1.377 (6)	C37—C38	1.380 (5)
C14—H14	0.9300	C38—H38	0.9300
C14—C15	1.392 (5)	C38—C39	1.374 (6)
C15—H15	0.9300	C39—C40	1.366 (6)
C16—C17	1.380 (5)	C39—C42	1.521 (6)
C16—C21	1.374 (5)	C40—H40	0.9300
C17—H17	0.9300	C40—C41	1.371 (5)
C17—C18	1.385 (6)	C41—H41	0.9300
C18—H18	0.9300	C42—H42A	0.9600
C18—C19	1.363 (6)	C42—H42B	0.9600
C19—H19	0.9300	C42—H42C	0.9600
C19—C20	1.361 (6)		
			
C1—O1—H1	109.5	C16—C21—H21	118.7
C24—O2—H2	109.5	C20—C21—C16	122.6 (4)
C22—N1—C2	122.3 (3)	C20—C21—H21	118.7
O1—C1—C2	107.4 (3)	N1—C22—H22	119.6
O1—C1—C10	106.2 (3)	N1—C22—C23	120.7 (3)
O1—C1—C16	110.7 (3)	C23—C22—H22	119.6
C10—C1—C2	113.9 (3)	C24—C23—C22	120.6 (3)
C10—C1—C16	108.4 (3)	C28—C23—C22	120.6 (3)
C16—C1—C2	110.2 (3)	C28—C23—C24	118.8 (3)
N1—C2—C1	106.1 (3)	O2—C24—C23	121.0 (3)
N1—C2—H2A	109.8	O2—C24—C25	118.2 (3)
N1—C2—C3	107.8 (3)	C23—C24—C25	120.8 (3)
C1—C2—H2A	109.8	C24—C25—C36	120.2 (3)
C3—C2—C1	113.5 (3)	C26—C25—C24	117.8 (3)
C3—C2—H2A	109.8	C26—C25—C36	122.0 (3)
C2—C3—H3A	109.1	C25—C26—H26	118.1
C2—C3—H3B	109.1	C25—C26—C27	123.8 (3)
H3A—C3—H3B	107.9	C27—C26—H26	118.1
C4—C3—C2	112.3 (3)	C26—C27—C29	121.3 (3)
C4—C3—H3A	109.1	C28—C27—C26	116.7 (3)
C4—C3—H3B	109.1	C28—C27—C29	122.0 (3)
C5—C4—C3	122.6 (4)	C23—C28—H28	118.9
C5—C4—C9	116.4 (4)	C27—C28—C23	122.2 (3)
C9—C4—C3	121.0 (3)	C27—C28—H28	118.9
C4—C5—H5	119.1	C30—C29—C27	120.9 (3)
C4—C5—C6	121.7 (4)	C30—C29—C34	117.0 (3)
C6—C5—H5	119.1	C34—C29—C27	122.1 (3)
C5—C6—H6	119.6	C29—C30—H30	119.3
C7—C6—C5	120.7 (5)	C31—C30—C29	121.4 (4)
C7—C6—H6	119.6	C31—C30—H30	119.3
C6—C7—H7	120.1	C30—C31—H31	119.3
C6—C7—C8	119.9 (5)	C30—C31—C32	121.5 (3)
C8—C7—H7	120.1	C32—C31—H31	119.3
C7—C8—H8	119.9	C31—C32—C35	120.8 (4)
C7—C8—C9	120.2 (5)	C33—C32—C31	117.6 (3)
C9—C8—H8	119.9	C33—C32—C35	121.6 (4)
C4—C9—C8	121.1 (4)	C32—C33—H33	119.4
C4—C9—H9	119.4	C32—C33—C34	121.2 (4)
C8—C9—H9	119.4	C34—C33—H33	119.4
C11—C10—C1	123.4 (3)	C29—C34—C33	121.4 (3)
C11—C10—C15	118.0 (3)	C29—C34—H34	119.3
C15—C10—C1	118.3 (3)	C33—C34—H34	119.3
C10—C11—H11	119.2	C32—C35—H35A	109.5
C10—C11—C12	121.6 (4)	C32—C35—H35B	109.5
C12—C11—H11	119.2	C32—C35—H35C	109.5
C11—C12—H12	120.0	H35A—C35—H35B	109.5
C13—C12—C11	120.0 (4)	H35A—C35—H35C	109.5
C13—C12—H12	120.0	H35B—C35—H35C	109.5
C12—C13—H13	120.2	C37—C36—C25	120.5 (3)
C14—C13—C12	119.5 (4)	C41—C36—C25	122.3 (3)
C14—C13—H13	120.2	C41—C36—C37	117.3 (4)
C13—C14—H14	119.7	C36—C37—H37	119.5
C13—C14—C15	120.5 (4)	C38—C37—C36	121.0 (4)
C15—C14—H14	119.7	C38—C37—H37	119.5
C10—C15—H15	119.9	C37—C38—H38	119.3
C14—C15—C10	120.2 (4)	C39—C38—C37	121.4 (4)
C14—C15—H15	119.9	C39—C38—H38	119.3
C17—C16—C1	121.5 (3)	C38—C39—C42	121.3 (5)
C21—C16—C1	122.1 (3)	C40—C39—C38	116.8 (4)
C21—C16—C17	116.4 (4)	C40—C39—C42	121.9 (5)
C16—C17—H17	119.4	C39—C40—H40	118.7
C16—C17—C18	121.2 (4)	C39—C40—C41	122.7 (4)
C18—C17—H17	119.4	C41—C40—H40	118.7
C17—C18—H18	119.7	C36—C41—H41	119.6
C19—C18—C17	120.6 (4)	C40—C41—C36	120.8 (4)
C19—C18—H18	119.7	C40—C41—H41	119.6
C18—C19—H19	120.6	C39—C42—H42A	109.5
C20—C19—C18	118.8 (5)	C39—C42—H42B	109.5
C20—C19—H19	120.6	C39—C42—H42C	109.5
C19—C20—H20	119.8	H42A—C42—H42B	109.5
C19—C20—C21	120.3 (4)	H42A—C42—H42C	109.5
C21—C20—H20	119.8	H42B—C42—H42C	109.5

### Hydrogen-bond geometry (Å, º)

**Table:**

*D*—H···*A*	*D*—H	H···*A*	*D*···*A*	*D*—H···*A*
O2—H2···N1	0.82	1.80	2.539 (4)	148
O1—H1···N1	0.82	2.46	2.763 (3)	103
